# Psychopathology and eating behaviour in people with type 2 diabetes referred for bariatric surgery

**DOI:** 10.1007/s40519-022-01502-7

**Published:** 2022-12-10

**Authors:** C. Pekin, M. McHale, M. Seymour, E. Strodl, G. Hopkins, D. Mitchell, G. J. Byrne

**Affiliations:** 1 Faculty of Medicine, Academy of Psychiatry, University of Queensland, Brisbane, QLD Australia; 2 Faculty of Medicine, Department of Endocrinology and Diabetes, Royal Brisbane & Women’s Hospital, University of Queensland, Brisbane, QLD Australia; 3 Department of Psychology, Royal Brisbane & Women’s Hospital and School of Psychology, University of Queensland, Brisbane, QLD Australia; 4 School of Psychology and Counselling, Queensland University of Technology, Brisbane, Australia; 5 Division of Surgery Metro North Hospital and Health Service, Royal Brisbane & Women’s Hospital, Brisbane, Australia; 6 Mental Health Service, Royal Brisbane & Women’s Hospital, Brisbane, Australia; 7 Centre for Clinical Research, University of Queensland, Brisbane, Australia

**Keywords:** Psychopathology, Bariatric surgery, Obesity, Eating disorders

## Abstract

**Purpose:**

Psychopathology and disordered eating behaviours are putative pre-operative risk factors for suboptimal outcomes post-bariatric surgery. Documented psychopathology prevalence rates vary in bariatric candidate samples. Further, less attention has been paid to vulnerable subgroups such as people with diabetes who might be at an elevated risk. For these reasons, this study aimed to investigate the rates of psychopathology and disordered eating in pre-surgical candidates with type 2 diabetes mellitus (T2DM).

**Methods:**

Participants were 401 consecutive patients from a state-wide bariatric surgery service for people with T2DM. Psychopathology was measured using multi-modal assessment including diagnostic interview and battery of validated questionnaires. The mean age of the sample was 51 years with a mean BMI of 46 kg/m^2^. The majority of the sample was female (60.6%), born in Australia (87%) and 18.2% identified as Aboriginal and/or Torres Strait Islander.

**Results:**

Rates of current psychopathology in this sample included: major depressive disorder (MDD; 16.75%), generalised anxiety disorder (GAD; 20.25%), insomnia (17.75%) and binge eating disorder (BED; 10.75%). There were no significant differences on measures between people who endorsed Aboriginal and/or Torres Strait Islander status compared to those who did not endorse. The mean total score on the BES was 21.82 ± 10.40 (range 0–39), with 8.2% of participants meeting criteria for severe binge eating. Presence of an eating disorder was not significantly associated with degree of glycemic compensation. Average emotional eating scores were significantly higher in this study, compared to reference samples. Significantly increased binge eating severity and emotional eating severity was revealed for people with T2DM and comorbid MDD, social anxiety and eating disorders. Binge eating severity was associated with GAD, food addiction, substance use disorders, and history of suicide attempt but not emotional eating severity.

**Conclusion:**

Amongst people with T2DM seeking bariatric surgery, MDD, GAD and emotional eating were common. Psychopathology in a sample of people with T2DM seeking bariatric surgery was significantly associated with severity of disordered eating. These findings suggest people with T2DM seeking bariatric surgery may be vulnerable to psychopathology and disordered eating with implications for early identification and intervention.

**Level of evidence:**

Evidence obtained from cohort or case–control analytic studies.

**Supplementary Information:**

The online version contains supplementary material available at 10.1007/s40519-022-01502-7.

## Introduction

The pre-surgical predictors and underlying mechanisms contributing to insufficient weight loss, early weight regain and poor diabetes control in people undergoing bariatric surgery are complex and poorly understood [1]. Some evidence suggests that psychopathology (including psychiatric disorders, disordered eating behaviour and substance use disorders) may provide an explanation as to why some patients experience sub-optimal results [2]. The relationship between psychopathology and type 2 diabetes mellitus (T2DM) is well established, with a bidirectional relationship supported [3–10]. Studies indicate psychopathology increases the risk of T2DM [5, 9, 11] and that a diagnosed eating disorder has a specific independent risk for the onset of T2DM [6, 12]. It is also well established that T2DM increases the risk of and is highly comorbid with mental health conditions [13].

Despite these strong relationships, there is inconclusive evidence for the specific pre-operative psychological predictors of bariatric surgery outcomes and scant evidence in people with T2DM. A recent scoping review of bariatric surgical candidates revealed the predisposing factors for weight regain were multifactorial and included depression, psychiatric conditions, binge eating disorder (BED) and loss of control eating [1]. In addition, disordered eating has been found to predict insufficient weight loss at 5 years in bariatric surgical patients [14]. The relevance of screening for psychopathology is also highlighted by the increased risk of suicidal ideation, non-suicidal self-injury and suicide attempts following bariatric surgery [13, 15, 16]. Evidence suggests that psychopathology predicts post-operative suicidal behaviour (e.g. history of suicidal ideation, history of major depressive disorder (MDD) [17] and current antidepressant use [18]). Accurate screening methods to identify these pre-operative risk factors are, therefore, paramount in facilitating timely identification and intervention.

There is sparse evidence examining psychopathology and eating behaviour in people with T2DM seeking bariatric surgery. Review of the literature yielded inconsistent definitions and measurement of comorbid T2DM and psychopathology [19–24] including primarily lifetime, not current measures of MDD and BED [23, 24]. One study examined the prevalence of concurrent BED and T2DM in bariatric surgery candidates, revealing 8.2% in their sample met criteria for both [19]. Considering the dearth of psychopathology research for people with T2DM undergoing bariatric surgery and the significant burden on patients if undetected, further research is warranted to explore the prevalence and risk of psychopathology in this specific cohort [19–24]. The current study aimed to explore rates of psychopathology and disordered eating using a multi-modal assessment strategy in an unselected group of patients referred for consideration of bariatric surgery for management of T2DM.

## Methods

### Participants

Study participants were 401 patients from a state-wide bariatric surgery service for people with T2DM. The study will refer to the cohort as the RB-BariPsy Cohort. The mean age of the participants was 51.14 years ± 9.48 (range 18–65 years) with a mean body mass index (BMI) of 46.23 ± 7.2 (range 30.5–69.3 kg/m2). Table 1 summarises their demographic and clinical characteristics. Of the participants, 18.2% identified as Aboriginal and/or Torres Strait Islander, 86.5% were born in Australia, 5.5% born in New Zealand, 2.5% born in England and 6.5% born in other countries.

**Table 1:** Demographic and clinical characteristics

Variable	Frequency *N* (%) Means ± SD (*N* = 401)
Sex (Female)	243 (60.6%)
Age (in years)	51.14 ± 9.48
ATSI	73 (18.2%)
BMI (kg/m2) at baseline	46.23 ± 7.2 (30.5 – 69.3)
Weight (kg) at baseline	131.42 ± 20.39
HbA1c (%)	8.69 ± 1.47
AST units	29.53 ± 17.18
ALT units	37.93 ± 21.33
Metformina	366 (91.3%)
Insulina	252 (62.8%)
Total daily dose insulin (units)	77.15 ± 142.24
GLP-1 agonist	109 (27.2%)
Referrer (endocrinologist) b	348 (86.8%)
Country of birth (Australia)	347 (86.5%)
Age of diabetes onset (in years)	39.56 ± 10

*Note* *ATSI* Person identifies as either Aboriginal and/or Torres Strait Islander; *BMI* body mass index; *HbA1c* haemoglobin A1c. *AST* aspartate aminotransferase; *ALT* alanine aminotransferase; *GLP-1 agonist* glucagon-like peptide-1 (GLP-1) agonists. **a **Number (%) of patients prescribed. **b** Number (%) of patients referred by consultant endocrinologist

## Procedure

The present study investigated a consecutive series of adults with T2DM seeking bariatric surgery from a public hospital service between 2017 and 2021 (labelled RB-BariPsy cohort). The service is a state-wide publicly funded service with the following inclusion criteria: adults between the ages of 18 and 65 years with T2DM and glycated haemoglobin (HbA1c) greater than 6.5% despite two or more anti-hyperglycaemic agents, weight less than 185 kg (407.86 pounds), BMI greater than 30 kg/m2. Psychosocial risk factors (Table 2), clinical measures and psychopathology measures were collected at baseline clinic appointment. Anthropometric data were measured at date of assessment and again at date of surgery.

**Table 2:** Psychosocial risk factors

Risk factor	Frequency
Childhood emotional neglect (*n* = 401)	129 (32.2%)
Childhood physical abuse (*n* = 401)	102 (25.4%)
Childhood sexual abuse (*n* = 401)	62 (15.5%)
Parental separation before 18 (*n* = 401)	72 (18%)
Completed 10 years of schooling or less (*n* = 399)	211 (52.88%)
No tertiary education (*n* = 399)	312 (77.8%)
Learning difficulties (*n* = 400)	53 (13.2%)
Unable to read and write (*n* = 400)	17 (4.2%)
Diagnosed intellectual impairment (*n* = 400)	28 (7%)
Bullied in childhood (*n* = 400)	164 (40.9%)
Unemployed (*n* = 400)	280 (70%)
Previous relationship history (*n* = 400)	321 (80%)
Currently single (*n* = 400)	197 (49.1%)
History of domestic violence (*n* = 400)	77 (19.2%)
Children (*n* = 400)	195 (48.6%)
Family psychiatric history (*n* = 400)	141 (35.2%)
Parental substance use in childhood (*n* = 399)	58 (14.5%)
Currently prescribed psychotropic medication (*n* = 400)	100 (24.9%)
Social support (high)	210 (52.4%)

### Ethical approval

The study was approved by the Human Research Ethics Committee (HREC) of the Royal Brisbane and Women’s Hospital (approval code LNR/2020/QRBW/65708). As the study employed deidentified data from a clinical database, individual patient consent was not required.

## Measures

Diagnostic Interview. Participants underwent semi-structured clinical interviews using an adapted version of the Structured Clinical Interview for DSM-5 (SCID-5, [25]). All interviews were conducted by clinical psychologists. Psychosocial risk factors were collected via structured clinical interview. Psychosocial risk factors were coded based on a protocol written for retrospective chart audit.

Yale Food Addiction Scale (YFAS). The YFAS is a 25-item self-report measure assessing food addiction, where food addiction is conceptualised as consuming larger amounts of food than intended despite repeated failed attempts at cessation [26]. The original measure has two scoring options: a symptom count of “addiction criteria” ranging 0–7 and a diagnosis of “yes/no”. Internal reliability, convergent validity and discriminant validity have been demonstrated as adequate [26]. Internal consistency in the RB-BariPsy cohort was acceptable (*α* = 0.76).

Dutch Eating Behaviour Questionnaire (DEBQ). The DEBQ is a 33-item self-report assessment with three main subscales measuring eating behaviour. These are labelled restrained eating (10 items), emotional eating (13 items) and external eating (10 items) and have been shown to exhibit good psychometric properties [27, 28]. The Emotional Eating subscale has two dimensions: diffuse emotions (eating in response to diffuse emotions [4 items]) and clearly labelled emotions (eating in response to clearly labelled emotions [9 items]) [27]. The restrained eating (*α* = 0.88), emotional eating (*α* = 0.954) and external eating (*α* = 0.87) subscales were found to have good internal consistency in the RB-BariPsy cohort. Further, good internal consistency was found in the RB-BariPsy for the clearly labelled emotions (*α* = 0.94) and diffuse emotions (*α* = 0.88) subscales.

Binge Eating Scale (BES). The BES consists of 16 self-report items measuring behavioural manifestations of binge eating and feelings and cognitions surrounding a binge episode [29]. Designed as a measure of severity, each item is rated on a Likert scale from 0 to 3. It has been demonstrated to be sensitive and specific in distinguishing between compulsive and normal eaters and validity in clinical populations [30, 31]. It is the most commonly used clinical assessment of binge eating in pre-bariatric assessment [32]. In the RB-BariPsy cohort, internal consistency was found to be good (*α* = 0.89).

Hospital Anxiety and Depression Scale (HADS). The HADS is a 14-item self-report measure with 7 items measuring anxiety and 7 items measuring depression [33]. It uses a 4-point (0–3) Likert severity scale, with 2 reverse scored items. It has demonstrated good internal consistency for both scales (0.80 for anxiety and 0.76 for depression; [34]). The HADS has been used to track trends in people with obesity seeking weight loss interventions [35]. The comparison sample was the Swedish Obesity Subjects [35] (*N* = 655). In the RB-BariPsy cohort internal consistency was good for the Anxiety subscale (*α* = 0.83) and acceptable for the Depression subscale (*α* = 0.71).

Alcohol Use Dependence Test (AUDIT). The AUDIT measures alcohol use with three domains: alcohol consumption/hazardous drinking, alcohol-related problems/harmful drinking and alcohol dependence symptoms [36]. It was designed to detect hazardous and harmful drinking on a spectrum, providing opportunity for early and targeted interventions. Compared to other alcohol screening tools, the AUDIT has adequate reliability, validity, sensitivity and specificity [37, 38]. Internal consistency in the RB-BariPsy cohort was acceptable (*α* = 0.76). King et al. [13] is a sample of bariatric candidates from the Longitudinal Assessment of Bariatric Surgery-2 study; (2458 participants, 78.8% female).

Grazing Questionnaire (GQ). The GQ is a seven-item self-report measure of grazing, an eating pattern characterised by repetitive eating of unplanned small amounts of food [39]. The GQ consists of two subscales: a grazing behaviour and a loss of control grazing scale. Internal consistency in this sample was good (*α* = 0.91). The grazing behaviour subscale (*α* = 0.87) and loss of control subscale (*α* = 0.91) demonstrated good internal consistency in the RB-BariPsy cohort.

## Statistical analyses

Descriptive statistics included counts (percentages) and means (standard deviations). Chi-squared test and ANOVA were used to compare the patient characteristics by group. Comparisons for the AUDIT, Grazing Questionnaire and DEBQ compared the current data to the comparison samples [13, 27, 39]. Pearson’s correlation was used to test for associations between insulin levels, HbAlC and disordered eating behaviours. Analyses were run in the IBM SPSS Statistics Package (version 26).

## Results

### Ethnicity

There were no statistically significant differences in psychosocial risk factors or in measures of psychopathology between those who were identified as Aboriginal and/or Torres Strait Islander and those who did not (Please see Table 6 in Supplementary). Psychosocial risk factor prevalence rates for all study participants are presented in Table 2.

## Frequency of psychopathology and eating behaviour

The mean total score on the BES was 21.82 ± 10.40 (range 0–39). Using the cut-off 27 used in the previous studies [29], 8.2% of participants met criteria for severe binge eating. Grazing behaviour mean total grazing score was 10.82 ± 5.9. This can be compared to the score of 18.6 ± 4.59 found in the validation study [39].

Prevalence rates of risk factors derived from the diagnostic interview are displayed in Table 3, indicating high endorsement of emotional eating, insomnia, anxiety-related conditions and reliance on beverages. On the HADS, depression mean was 6.24 ± 4.13, with 28.4% of the sample meeting caseness criteria. For HADS, anxiety mean was 6.79 ± 4.12 with 39.1% meeting caseness [33]. Table 4 displays the DEBQ data, indicating that external eating, emotional eating (diffuse emotions and clearly labelled emotions) were significantly different to both reference samples. AUDIT data are presented in Table 5 displaying that alcohol consumption, total score, dependence symptoms and alcohol-related harm were significantly lower than the comparison sample [13].

**Table 3:** Interview-based psychopathology and relationship with disordered eating

Psychopathology	Lifetime (*N* = 400)^a^ *N (*%)	Current (*n* = 400)^a^ *N (*%)	Emotional eating (DEBQ)	Binge eating (BES)
Mood				
Major depressive disorder	193 (48.25%)	67 (16.75%)	*F*(3, 358) = 5.82**	*F*(3,371) = 3.6*
Bipolar affective disorder	7 (1.75%)	2 (0.5%)	*F*(2, 359) = 1.07	*F*(2,372) = 2.00
Anxiety-related				
Generalised anxiety disorder	102 (25.5%)	81 (20.25%)	*F*(3,358) = 2.07	*F*(3,371) = 3.82*
Panic disorder	66 (16.5%)	22 (5.50%)	*F*(2,359) = 2.56	*F*(2,372) = 2.16
Social anxiety disorder	63 (15.75%)	49 (12.25%)	*F*(3,358) = 3.26*	*F*(3,371) = 4.64*
Agoraphobia	38 (9.5%)	20 (5%)	*F*(2,359) = 4.24*	*F*(2,372) = 4.72*
Post-traumatic stress disorder	77 (19.25%)	25 (6.25%)	*F*(3,358) = 1.51	*F*(3,371) = 1.05
Obsessive compulsive disorder	7 (1.75%)	3 (0.75%)	*F*(2,359) = 0.70	*F*(2,372) = 1.2
Anorexia nervosa	9 (2.25%)	0 (0)	^b^	^b^
Bulimia nervosa	8 (2%)	1 (0.25%)	*F*(2,359) = 1.92	*F*(2,372) = 5.38*
Binge eating disorder	69 (17.25%)	43 (10.75%)	*F*(3,358) = 8.5**	*F*(3,371) = 19.72**
Number of binge eating episodes per week	0.4 ± 1.44	0.4 ± 1.44		
Night eating syndrome	46 (11.5%)	33 (8.25%)	*F*(3,358) = 2.79*	*F*(3,371) = 3.00*
Emotional eating	120 (30%)	93 (23.25%)	*F*(2,359) = 14.93**	*F*(2,373) = 5.55*
Other specified feeding and eating disorder	62 (15.5%)	47 (11.7%)	*F*(3,358) = 5.95**	*F*(3,371) = 5.37**
Food hoarding	3 (0.75%)	0 (0%)	*F*(1,360) = 2.35	*F*(1,373) = 0.003
Food addiction	18 (4.5%)	16 (4%)	*F*(3,358) = 1.99	*F*(3,371) = 2.65*
Reliance on beverages	57 (14.25%)	58 (14.5%)	*F*(8,353) = 1.59	*F*(8,366) = 0.56
History of attempted suicide	43 (10.75%)	0 (0%)	*F*(1,360) = .06	*F*(1,373) = 4.53*
History of deliberate self-harm	11 (2.75%)	1 (0.25%)	*F*(2,359) = 1.22	*F*(2,372) = .514
Alcohol use disorder	72 (18%)	13 (3.25%)	*F*(3,358) = 0.49	*F*(3,371) = 0.22
Cannabis use disorder	51 (12.75%)	2 (0.5%)	*F*(3,358) = 0.94	*F*(3,371) = 3.06*
Tobacco use	136 (34%)	5 (1.25%)	*F*(2,359) = 2.07	*F*(2,372) = 6.13*
Other substances	23 (5.75%)	1 (0.25%)	*F*(2,359) = 1.17	*F*(2,372) = 0.26
Hoarding	6 (1.5%)	4 (1%)	^b^	^b^
Skin picking	4 (1%)	2 (0.5%)	^b^	^b^
Psychosis	9 (2.25%)	1 (0.25%)	^b^	^b^
Personality disorder diagnosis history	23 (5.75%)	2 (0.5%)	^b^	^b^
History of mental health admission	34 (8.5%)	0 (0%)	*F*(1,360) = 0.69	*F*(1,373) = 7.7*
Insomnia		71 (17.75%)	*F*(1,360) = 1.06	*F*(1,373) = 1.92

*Note* Tobacco current use, recent suicide attempt (in past 6 months) are both exclusion criteria on the service referral form. One way ANOVAs were used. * Significant at *p* < .05 level. ** Significant at *p* < .001

^a^ 1 missing, *n* = 400

^b^ Analyses not conducted due to less than six in each group

**Table 4:** Comparison of eating behaviours with reference groups

	Current sample			Reference sample	
Eating pattern	*M* ± *SD* *(N* = 401)	HbAlC *r*	Insulin dose *r*	all in reference group [27] (*N* = 120) *M* ± *SD*	Obese sample [27] (*N* = 91) *M* ± *SD*
Restrained eating	2.64 ± 0.76	0*.*14	.05	2.21 ± 0.92**	2.66 ± 0.86
Emotional eating	2.45 ± 0.98	0.00	−.09	1.62 ± 0.6) **	2.11 ± 0.73**
External eating	2.91 ± 0.74	0.11	−.03	2.66 ± 0.54**	2.71 ± 0.58*
Clearly labelled emotions	2.35 ± 1.01	0.02	−.07	1.76 ± 0.68**	1.97 ± 0.76**
Diffuse emotions	2.68 ± 1.18	0.05	−.09	2.28 ± 0.82**	2.42 ± 0.85*

*Note* [27] is the sample used to develop the original DEBQ scale (120 participants: 80 female). Independent samples *t* tests were used to compare to reference groups. Correlation analysis compared HbA1C and insulin dose to eating patterns. * Significant at *p* < .05 level. ** Significant at *p* < .001

Units of measurement

**Table 5:** Comparison of alcohol use pre-surgery with reference group

AUDIT Items	Current sample prevalence *n* (%)	Reference sample [13] *n* = 1400
AUDIT consumption at hazardous level ≥ 8	6 (1.4%)**	*n* = 1357 266 (19.6%)
AUDIT score ≥ 8	25 (6.2%)**	36 (2.6%) *n* = 1392
Alcohol dependence symptoms	2 (0.6%)*	39 (2.8%) *n* = 1391
Alcohol-related harm	1 (0.3%)**	94 (6.8%) *n* = 1391
Alcohol use disorder	26 (6.5%)	106 (7.6%) *n* = 1389

Note. Alcohol Use Disorder defined as AUDIT score ≥ 8 or indication of alcohol dependence symptoms or alcohol-related harm. * Significant at *p* < .05 level. ** Significant at *p* < .001

King et al. [13] is a sample of bariatric candidates from the Longitudinal Assessment of Bariatric Surgery-2 study; (2458 participants, 78.8% female). Compared using Chi-squared analysis

## Presence of eating disorders and degree of glycemic compensation

There was no difference in baseline HbA1C between participants diagnosed with BED (*F*(3, 396) = 0.23, p = 0.87], BN (*F*(3, 397) = 0.11, *p* = 0.9], NES (*F*(3, 396) = 0.49, *p* = 0.69], OSFED (*F*(3, 396) = 1.89, *p* = 0.13] and those with historical or no diagnosis. At baseline, total daily dose of insulin was not significantly correlated with binge eating severity *r* = -0.09, *p* = 0.07. There was no significant difference in total daily insulin dose between groups identified with BED and those with historical or no BED diagnosis (*F*(3, 394) = 1.42, *p* = 0.24).

## Discussion

To our knowledge, this is the first study to utilise a multi-modal assessment method to measure psychopathology and disordered eating in people with T2DM seeking bariatric surgery. Self-reported baseline emotional and binge eating were found to be significantly increased in those diagnosed with MDD, social anxiety disorder, agoraphobia, BED, NES, OSFED and those without diagnoses. In contrast, a current diagnosis of GAD, food addiction, tobacco use disorder, cannabis use disorder, historical mental health admission and suicide attempt history were only associated with binge eating. These associations suggest eating behaviour may be associated with affect regulation in the presence of clinical levels of social anxiety and depression for people with T2DM. Further research is warranted to test whether these associations exist post-operatively and the risks of disordered eating on surgical outcomes if these psychological conditions remain untreated. Given the association with substance use disorders and historical suicide attempts on binge eating severity alone, it appears relevant to include both a binge eating and emotional eating scale to accompany clinical interview in this group to detect the severity of pre-operative disordered eating. Accordingly, this finding may have implications for distinct causal mechanisms of disordered eating which requires further research to explore. Alternatively, this may reflect endorsing behaviour from patients with these conditions, e.g. patients may have insight into the behaviours of their conditions such as cannabis or tobacco use however do not attribute affect-based reasons for these.

Rates of adverse childhood events (ACEs) were comparable to other pre-bariatric studies [44]. Previous studies highlight the association between childhood trauma and risk of both psychopathology and obesity [45, 46], as well as the possible role of past interpersonal trauma as a risk factor for poor outcome in bariatric surgery [47].

Rates for depression and anxiety were consistent across self-report and diagnostic interview measures. Accurate identification at baseline is essential given that the current anxiety-related disorders have been found to predict less BMI reduction, weight loss and percent excess weight loss 5 years post-bariatric surgery [2].

Lifetime rates of eating disorders were also comparable to general bariatric studies [41, 43] highlighting the need for collection of history in assessment and intervention in this sample. Inconsistent prevalence rates have been reported for BED in general bariatric surgical patients (e.g. 3.4%, [40], 16%; [41], 6.7%, [42], 23.3% [48]). The current sample had lower levels of BED compared to Mitchell et al. [43], which included a subset of patients with T2DM (34% of their sample with T2DM met criteria for BED), but comparable levels to that of Webb et al. [19] who found that 8.2% of people with T2DM in their sample met criteria for BED.

Rates of emotional eating were significantly elevated compared to the reference groups[27]. Of note, rates of eating in response to clearly labelled emotions and in response to diffuse emotions were significantly elevated compared to reference samples. Given evidence that emotional eating is predictive of a failure to achieve expected weight loss in general bariatric surgery patients [49], this highlights the need for ongoing screening of emotional eating in this population. Further intervention or adjuvant treatments may need to be considered for the subgroup of candidates in this sample who endorse emotional eating.

Insomnia was common in this sample and the prevalence rate of night eating syndrome (NES) was comparable to previously documented studies [50]. Further research is warranted to explore insomnia and NES in this cohort given its association with substandard glycemic control in prior research [51]. History of attempted suicide was also commonly endorsed compared to the Australian population (0.25%; [52]) and psychotropic medication was commonly prescribed. Whilst there could be multiple explanations, taken together these findings indicate a vulnerable sample present with multiple risk factors. These specific risks are of particular importance in this group as prior studies have suggested the association with suicidal behaviour post-bariatric surgery [17].

On the AUDIT, measures of alcohol consumption, total use, dependence symptoms and alcohol-related harm were significantly lower than the reference sample of general bariatric surgical candidates [13]. This finding was discrepant to the rates found on clinical interview. This highlights the need for further research examining the reliability and validity of alcohol assessment pre-surgery in the context of people seeking surgical management of T2DM. Previous studies have highlighted the role of demand characteristics such as impression management in pre-surgical candidates [53]. This may provide provisional evidence for the use of multi-modal assessment pre-surgery to effectively screen for harmful levels of alcohol consumption in this cohort, especially given that high alcohol consumption post-bariatric surgery can severely impact quality of life [54].

## What is already known on this subject?

There is sparse evidence examining psychopathology and eating behaviour in people with T2DM seeking bariatric surgery.

## What does this study add?

Amongst people with T2DM seeking bariatric surgery, those diagnosed with MDD, social anxiety disorder, agoraphobia, BED, NES and OSFED endorsed increased levels of emotional and binge eating compared to those without these conditions. In contrast, those with a current diagnosis of GAD, food addiction, tobacco use disorder, cannabis use disorder and historical mental health admission and suicide attempt history endorsed greater binge eating scores. These findings suggest people with T2DM seeking bariatric surgery may be vulnerable to psychopathology and disordered eating with implications for early identification and intervention.

## Limitations

Limitations to this study include the retrospective cross-sectional design and absence of a control group. Demand characteristics may also be present such as social desirability bias in patients awaiting bariatric surgery.

## Future research

Further investigation of reliable and valid methods for the early identification and intervention for psychopathology and disordered eating in this subgroup is warranted. Longitudinal designs exploring the course of psychopathology and disordered eating, including trajectories to identify vulnerable patient groups would be advantageous.

## Conclusions

Amongst people with T2DM seeking bariatric surgery, those diagnosed with current pre-operative depression, social anxiety and eating disorders endorsed greater severity on measures of both binge and emotional eating. In contrast, those with a current diagnosis of GAD, food addiction, substance use disorders and suicide attempt history endorsed greater binge eating but not emotional eating. There were no significant differences on any measured disordered eating or psychopathology between those who endorsed Aboriginal and/or Torres Strait Islander status and those who did not. These findings suggest people with T2DM seeking bariatric surgery may be vulnerable to psychopathology and disordered eating with implications for early identification and intervention.

## Supplementary Information

Below is the link to the electronic supplementary material.

Supplementary file1 (DOCX 28 kb)

## Data Availability

The datasets generated during and/or analysed during the current study are available from the corresponding author on reasonable request.
