# Supplementary material for: Psychopathology and eating behaviour in people with type 2 diabetes referred for bariatric surgery
Source: Eat Weight Disord. 2022 Dec 10;27(8):3627–35. doi: 10.1007/s40519-022-01502-7 (PMC9803743; doi:10.1007/s40519-022-01502-7)
Supplement: Supplementary file 1 — Supplementary file1 (DOCX 28 kb) [file 40519_2022_1502_MOESM1_ESM.docx]

Supplementary Material

**Table 6.** Psychopathology Comparison on People who Self-endorsed Aboriginal and/or Torres Strait Islander Status

| **ANOVA** | | | | | | |
| --- | --- | --- | --- | --- | --- | --- |
|  | | Sum of Squares | df | Mean Square | F | Sig. |
| Analysis Binge Eating Summary Score pre | Between Groups | 47.243 | 1 | 47.243 | .644 | .423 |
|  | Within Groups | 27728.997 | 378 | 73.357 |  |  |
|  | Total | 27776.239 | 379 |  |  |  |
| DEBQ Restrained Scale pre | Between Groups | .956 | 1 | .956 | 1.652 | .200 |
|  | Within Groups | 211.912 | 366 | .579 |  |  |
|  | Total | 212.868 | 367 |  |  |  |
| DEBQ Emotional Scale pre | Between Groups | .234 | 1 | .234 | .241 | .624 |
|  | Within Groups | 358.496 | 369 | .972 |  |  |
|  | Total | 358.730 | 370 |  |  |  |
| DEBQ External Scale pre | Between Groups | .018 | 1 | .018 | .034 | .855 |
|  | Within Groups | 204.636 | 371 | .552 |  |  |
|  | Total | 204.655 | 372 |  |  |  |
| DEBQ Clearly Labelled Emotion Scale pre | Between Groups | .250 | 1 | .250 | .244 | .622 |
|  | Within Groups | 371.968 | 363 | 1.025 |  |  |
|  | Total | 372.219 | 364 |  |  |  |
| DEBQ Diffuse Emotions Scale pre | Between Groups | .784 | 1 | .784 | .565 | .453 |
|  | Within Groups | 509.310 | 367 | 1.388 |  |  |
|  | Total | 510.094 | 368 |  |  |  |
| GQ Grazing Behaviour pre | Between Groups | 1.013 | 1 | 1.013 | .084 | .772 |
|  | Within Groups | 2758.650 | 229 | 12.047 |  |  |
|  | Total | 2759.662 | 230 |  |  |  |
| GQ Loss of Control Grazing pre | Between Groups | 1.446 | 1 | 1.446 | .176 | .675 |
|  | Within Groups | 1875.428 | 228 | 8.226 |  |  |
|  | Total | 1876.874 | 229 |  |  |  |
| GQ total pre | Between Groups | .022 | 1 | .022 | .001 | .980 |
|  | Within Groups | 7948.961 | 226 | 35.172 |  |  |
|  | Total | 7948.982 | 227 |  |  |  |
| AUDIT Total pre | Between Groups | 1.682 | 1 | 1.682 | .188 | .665 |
|  | Within Groups | 2527.065 | 282 | 8.961 |  |  |
|  | Total | 2528.746 | 283 |  |  |  |
| Alcohol Consumption pre | Between Groups | .101 | 1 | .101 | .027 | .871 |
|  | Within Groups | 1082.000 | 284 | 3.810 |  |  |
|  | Total | 1082.101 | 285 |  |  |  |
| Alcohol Dependence pre | Between Groups | 4.177 | 1 | 4.177 | 3.633 | .058 |
|  | Within Groups | 326.592 | 284 | 1.150 |  |  |
|  | Total | 330.769 | 285 |  |  |  |
| Alcohol Related Problems pre | Between Groups | .119 | 1 | .119 | .126 | .723 |
|  | Within Groups | 270.829 | 287 | .944 |  |  |
|  | Total | 270.948 | 288 |  |  |  |
